# Patient-derived organoids guide personalized therapy for KRAS-mutant pancreatic cancer: synergistic MEK/mTOR inhibition and predictive chemotherapy responses

**DOI:** 10.3389/fimmu.2026.1760379

**Published:** 2026-05-15

**Authors:** Xiaorui Wang, Lijuan Liu, Feng Li, Yanyan Da, Fang Hu, Xin Qi, Yiwen Pan, Meng Jiang, Peng Hou, Jin Yang

**Affiliations:** 1 Department of Precision Medicine Center, The First Affiliated Hospital of Xi’an Jiaotong University, Xi’an, China; 2 Department of Endocrinology, Oncology, The First Affiliated Hospital of Xi’an Jiaotong University, Xi’an, China; 3 Department of Anesthesiology, The First Affiliated Hospital of Xi’an Jiaotong University, Xi’an, China; 4 Department of Medical Oncology, The First Affiliated Hospital of Xi’an Jiaotong University, Xi’an, China

**Keywords:** NGS, PDAC, PDO, PDX, personalized medicine, dual-drug combination

## Abstract

**Introduction:**

Pancreatic ductal adenocarcinoma (PDAC) is an aggressive malignancy driven by KRAS mutations in ~90% of cases, with high heterogeneity and limited efficacy of single targeted agents. Patient-derived organoids (PDOs) and xenografts (PDXs) offer promising platforms for personalized therapy by replicating tumor characteristics.

**Methods:**

We established a PDAC biobank with 69% PDO and 31% PDX success rates from 66 patient samples. Next-generation sequencing (NGS) of 425 oncogenes was performed, followed by 32-drugs in vitro screening in PDOs. The synergistic effects of the MEK inhibitor trametinib combined with the mTOR inhibitor AZD8055 or the pan-CDK inhibitor flavopiridol were evaluated in PDOs and validated in matched PDXs. We also validated PDOs in predicting clinical gemcitabine/paclitaxel (Gem/PTX) responses.

**Results:**

PDOs preserved tumor histological and genetic feature, with consistent drug responses across early and late passages. Trametini/AZD8055 exerted robust synergistic antitumor effects in all tested PDO and PDX models, while trametinib/flavopiridol failed in PDO/PDX-099. The Gem/PTX regimen achieved 75 -95% growth inhibition in PDOs, and the in vitro results were highly consistent with the in vivo efficacy in PDXs and the clinical CA19-9 remission of patients.

**Discussion:**

This study effectively integrated two preclinical models, PDOs and PDXs, both in vitro and in vivo, which are highly regarded in the fields of drug discovery and personalized medicine. The trametinib/AZD8055 combination is a promising precision therapeutic strategy, and PDOs can serve as a reliable tool to guide clinical therapy selection. Despite limitations such as small sample size, lack of tumor microenvironment and immune components in the model system, this work provides important preclinical evidence for the clinical translation of PDOs in the personalized therapy of PDAC.

## Introduction

Pancreatic ductal adenocarcinoma (PDAC) remains one of the most devastating and lethal malignancies worldwide, clinically characterized by late-stage presentation at diagnosis, high invasiveness, rapid disease progression, and significant resistance to most conventional therapeutic regimens. Recent clinical statistics indicate that the overall 5-year survival rate of PDAC is approximately 13%, a figure that underscores the urgent clinical need for more effective diagnostic tools, therapeutic strategies, and predictive models (1–3).

A significant challenge in treating PDAC is its heterogeneity, which results in variable therapeutic responses among patients. There is a lack of data to predict responses to targeted therapies in PDAC patients due to the absence of reliable biomarkers (4–6). The oncogenic mutation of KRAS serves as a key driver of disease initiation and maintenance in approximately 90% of cases, leading to the persistent activation of KRAS. This aberrant activation of the KRAS gene engages multiple downstream pathways and cellular processes (7–9). Strategies have often focused on developing agents that target associated effector pathways. While targeted therapies based on next-generation sequencing (NGS) have been implemented in clinical settings, selecting effective targeted agents remains a formidable challenge, underscoring the necessity for a “personalized” approach in the application of these therapies (10–14).

Patient-derived organoid (PDO) and xenograft (PDX) models more accurately represent primary tumors due to the extensive heterogeneity of their constituent cancer cells. These models are increasingly recognized as vital preclinical platforms to investigate the molecular landscape of cancer, including applications in personalized drug screening and the assessment of drug safety (15).

In this study, we established an organoid biobank with a success rate of approximately 70%. Additionally, PDX models corresponding to the tumor organoids were generated. To enhance the reliability of our preclinical data, we conducted a comprehensive study with the following objectives: (1) establish PDO and PDX models for individual patients, (2) perform NGS on primary tumor tissues and the established preclinical models to identify targeted gene mutations, (3) conduct drug screening *in vitro* using PDO models, (4) validate in PDX models, and (5) further validate efficacy combined with patient clinical responses. Through multi-dimensional optimization of the study design, we systematically elucidated the efficacy characteristics of the precision combination of next-generation MEK/mTOR inhibitors in PDAC, filling the knowledge gaps of previous studies in personalized combination screening strategies, validation with clinically relevant models, and optimization of pathway intervention mechanisms. This study provides more clinically relevant experimental evidence and strategic references for the targeted therapy of KRAS-mutant PDAC.

## Materials and methods

### Materials

#### Key resource table

**Table:** 

Reagent and resource	Source	Catalog
Antibodies		
KI67 rabbit polyclonal antibody	Proteintech	27309-1-AP
Mucin 5AC mouse monoclonal antibody	Abcam	ab24070
PDX1 rabbit monoclonal antibody	Abcam	ab219207
SOX9 rabbit monoclonal antibody	Abcam	ab185230
Anti-cytokeratin 7 mouse mAb	Servicebio	GB12225
重组Anti-cytokeratin 20抗体	Abcam	ab76126
Rabbit polyclonal anti-CDX2	Novus	NB100-2136
P53 antibody	Proteintech	60283-2-Ig
﻿PhosphoPlus^®^ p44/42 MAPK (Erk1/2) (Thr202/Tyr204) antibody duet	Cell Signaling Technology	8201S
﻿PhosphoPlus^®^ MEK1/2 (Ser217/Ser221) antibody duet	Cell Signaling Technology	8200S
Cyclin D1 (E3P5S) rabbit mAb	Cell Signaling Technology	55506S
S6 ribosomal protein rabbit mAb	Cell Signaling Technology	2217S
Phospho-S6 ribosomal rabbit mAb	Cell Signaling Technology	4858S
Phospho-RB-S780 rabbit mAb	Abclonal	AP0117
Cyclin A2 rabbit pAb	Abclonal	A7632
GAPDH monoclonal antibody	Proteintech	60004-1-Ig
﻿Peroxidase-AffiniPure goat anti-mouse IgG (H+L)	Jackson	115-035-003
﻿Peroxidase-AffiniPure goat anti-rabbit IgG (H+L)	Jackson	111-035-003
Chemicals, peptides, and recombinant proteins		
Advanced DMEM/F-12	Gibco	12634010
GlutaMAX™ supplement, 100×	Gibco	35050061
HEPES (1 M)	Gibco	15630080
Penicillin–streptomycin (P/S)	HyClone	SV30010
Phosphate-buffered saline (PBS)	HyClone	SH30256.01
Primocin™	Invitrogen	ant-pm-1
N2 supplement (100×)	Gibco	17502048
B-27™ supplement (50×)	Gibco	12587010
Nicotinamide	Sigma-Aldrich	N0636-100G
N-Acetyl-L-cysteine	Sigma-Aldrich	A9165-5G
RSPO1 protein, human, recombinant	Sino Biological	11083-HNAS
Human EGF	Peprotech	AF-100-15
Noggin protein, human, recombinant	Sino Biological	10267-HNAH
Gastrin I	Sigma-Aldrich	05-23-2301
FGF10 protein, human, recombinant	Sino Biological	10573-HNAE
Prostaglandin E2	Tocris	2296-10mg
RHOK inhibitor Y-27632	Abmole Bioscience	M1817
A-83-01	Tocris	2939/10
Forskolin	Tocris	1099
Collagenase	Sigma	C9407
Dispase II	Solarbio	D6430
DNase I	Roche	11284932001
Collagenase II	Solarbio	C8150
TrypLE™ Express (1X)	Gibco	12605010
Recovery cell culture freezing medium	Gibco	12648010
Cell recovery solution	Corning	354253
GFR matrigel	Corning	354230
Enhanced DAB Plus Kit	MXB Biotechnologies	DAB-2032
Neutral balsam	MXB Biotechnologies	DAB-0033
Modified hematoxylin solution	MXB Biotechnologies	CTS-1096
EDTA antigen retrieval solution	MXB Biotechnologies	MVS-0099
Elivision™super HRP (mouse/rabbit) IHC	MXB Biotechnologies	KIT-9922
Endogenous peroxidase blocking solution	MXB Biotechnologies	SP-KIT-A3
Normal goat serum for blocking	Zhongshan Jinqiao	ZLI-9056
Fetal bovine serum	BI	C04001-050
DMEM/high glucose	HyClone	SH30022.01
Neutral buffered formalin	Solarbio	G2161
CellTiter-Glo 3D reagent	Promega	G9681
Drugs/inhibitors		
Gemcitabine	Selleck	95058-81-4
Oxaliplatin (S1224)	Selleck	61825-94-3
Irinotecan (S1198)	Selleck	97682-44-5
5-Fluorouracil (S1209)	Selleck	51-21-8
Paclitaxel	Selleck	S1150
Capecitabine	Selleck	S1156
Docetaxel	Selleck	S1148
AZD0156	Selleck	S8375
Olaparib	Selleck	S1060
AZD1390	Selleck	S8680
Trametinib	Selleck	S2673
GW5074	Selleck	S2872
RAF709	Selleck	S8690
TAK-632	Selleck	S7291
SB590885	Selleck	S2220
Palbociclib	Selleck	S1116
HLM006474	Selleck	S8963
Flavopiridol	Selleck	S1230
Ribociclib	Selleck	S7440
Gefitinib	Selleck	S1025
Erlotinib	Selleck	S7786
Afatinib	Selleck	S1011
Neratinib	Selleck	S2150
APR-246	Selleck	S7724
COTI-2	Selleck	S8580
Taselisib	Selleck	S7103
Alpelisib	Selleck	S2814
Buparlisib	Selleck	S2247
MK-2206	Selleck	S1078
Everolimus	Selleck	S1120
AZD8055	Selleck	S1555
Temsirolimus	Selleck	S1044

### Methods

#### Human specimens

All tissues were obtained from patients who received surgical resection or fine-needle biopsy (FNB) at the First Affiliated Hospital of Xi’an Jiaotong University. Written informed consent was obtained from the donors for the research use of their clinical data and surgical specimens in this study. The studies were conducted in accordance with recognized ethical guidelines and was approved by the Ethics Committee of the First Affiliated Hospital of Xi’an Jiaotong University (approval number: XJTU1AF2024LSYY-320).

#### Human pancreatic tumor and normal organoid culture and analysis

For human tumor samples, tissues were minced and digested with collagenase II (5 mg/mL) in basal medium at 37 °C for a maximum of 1.5 h. Following digestion, the cell suspensions were then filtered through a 100-μm nylon cell strainer and centrifuged for 5 min at 1,500 rpm. The pellet was washed in cold Advanced DMEM/F12 and then were embedded in GFR matrigel. The gels were allowed to solidify at 37°C for 30 min and cultured in human complete medium (HPO): Advanced DMEM/F12, HEPES—10 mM, Glutamax—1×, penicillin/streptomycin—1×, primocin—1×, B27—1×, A83-01—500 nM, hEGF—50 ng/mL, Noggin—100 ng/mL, hFGF10—100 ng/mL, R-spondin1—0.7 μg/mL, hGastrin I—0.01 μM, N-acetylcysteine—1.25 mM, nicotinamide—10 mM, PGE2—1 μM, FSK—1 μM, and Y27632—10 μM.

For normal samples, tissues were processed as detailed above, except that digestion was done by using collagenase (0.125 mg/mL), Dispase II (0.125 mg/mL), and DNase I (0.1 mg/mL) for a maximum of 1.5 h in wash buffer. After the GFR matrigel had solidified, the cells were cultured in human complete medium with complete media (HPO).

The culture media were replaced every 3 to 4 days. For serial passaging of organoids, these were treated with TrypLE solution for 10 min at 37°C. After digestion, an appropriate volume of basal medium was added (Advanced DMEM/F12, HEPES—10 mM, Glutamax 1×, penicillin/streptomycin 1×) to stop the digestion. The culture was centrifuged at 4°C at 1,500 rpm for 5 min and then embedded in the GFR matrigel. For storage, the organoids were dissociated and resuspended in recovery cell culture freezing medium and frozen according to standard procedures.

#### Histopathology and immunohistochemistry staining

Tissues were fixed in neutral buffered formalin and organoids were fixed in 4% paraformaldehyde, followed by dehydration, paraffin embedding, and slicing to a thickness of 5 µm. All slices were dried at 65 °C for 90 min. H&E staining or immunohistochemistry was performed according to standard protocols. For IHC, tumor tissues were deparaffinized with xylene and dehydrated with decreasing concentrations of ethanol. The endogenous peroxidase activity was blocked with 3% hydrogen peroxide. Microwave antigen retrieval technique was used. Nonspecific antigen sites were blocked using normal goat serum for 20 min at room temperature. The antibodies used for staining organoids and tissues were as follows: anti-PDX1 (1:500, ab185230, abcam), anti-SOX9 (1:2,000, ab185230, abcam), anti-MUC5AC (1:100, ab24070, abcam), anti-KI67 (1:250, ab92742, abcam), anti-CK7 (1:400, GB12225, Servicebio), anti-CK20 (1:100, ab76126, abcam), and anti-CDX2 (1:800, NB100-2136, Novus).

#### Next-generation sequencing—425-gene panel

DNA panel sequencing was conducted by Geneseeq Technology, Inc. Hybridization-based target enrichment was carried out with the GeneseeqOne™ pancancer gene panel (425 cancer-relevant genes, Geneseeq Technology, Inc.) and xGen Lockdown Hybridization and Wash Reagents Kit (Integrated DNA Technologies). The concentration of the captured libraries was quantified by quantitative polymerase chain reaction (qPCR) and loaded on the HiSeq4000 platform (Illumina).

#### Organoid viability assay

PDOs were seeded in 10 μL of GFR Matrigel matrix droplets in white, clear-bottom 96-well plates (4,000 cells per well) for 4 days and then treated with culture medium containing different drugs as well as DMSO control. Cell viability was detected using CellTiter-Glo 3D reagent according to the manufacturer’s protocol. The IC50 values were normalized to a *z*-score using the formula *z* = (*x* − μ)/σ, where *x* is the IC50 value of the PDO of interest, μ is the average IC50 for all PDOs tested, and σ is the standard of the IC50 values for all PDOs tested. All calculations were performed with these values to visualize differences in drug response between PDOs. Bliss synergy scores were calculated using SynergyFinder (http://www.synergyfinder.org/). SynergyFinder calculates a synergy score based on Bliss model (16, 17).

#### Western blot analysis

The human pancreatic cancer organoids were harvested with Cell Recover Solution and washed with ice-cold PBS. Cells were lysed in RIPA buffer containing protease and phosphatase inhibitors. The concentration of proteins in the cell lysates was quantified by using BCA Protein Assay Kit. Standard immunoblotting procedures were followed. The PVDF membranes were blocked in 5% BSA diluted in TBST for 1 h. Primary antibodies were incubated overnight at 4°C, namely: anti-MEK1/2 (1:1,000), phosphorylated MEK1/2 (1:1,000), ERK1/2 (1:1,000), phosphorylated ERK1/2 (1:1,000), cyclinA2 (1:1,000), cyclinD1 (1:1,000), phosphorylated RB (1:100), S6 (1:1,000), phosphorylate S6 (1:1,000), and GAPDH (1:5,000). After incubation with the primary antibody, the membranes were washed with 1× TBST three times for 30 min. The membranes were incubated with goat anti-rabbit or anti-mouse IgG secondary antibodies (1:5,000 dilutions) for 1.5 h at room temperature. The membranes were washed three times for 30 min and then visualized using a chemiluminescence assay.

ImageJ was used for densitometry, and the relative protein expression levels were analyzed by two-way ANOVA, followed by Sidak’s multiple comparisons test, to compare differences among four treatment groups within each protein. Data are presented as mean ± standard error of the mean (SEM). A *p*-value <0.05 was considered statistically significant.

#### *In vivo* experiments

Six- to 8-week-old female NCG mice were purchased from Jicuiyaokang company. The mice were housed and maintained in laminar flow cabinets under a specific pathogen-free environment in accordance with the current standards and regulations of the government. To construct pancreatic cancer patient-derived xenograft (PDX) models, the resected patient tumor tissue was cleaned, trimmed, and cut into millet grain-sized fragments (estimated 1 to 2 mm³, suitable for trocar implantation to ensure survival and growth). The tissue was transplanted subcutaneously into the mice to establish passage 0 (P0), which was passage to P1 (≈1.5 cm³ tumor volume, 2–4 months) and further to P2 (1 to 2 months) due to the insufficient number of P1 mice for the experiments. Compared with P0 and P1, P2 had faster growth and higher stability, ideal for pancreatic cancer pharmacodynamic evaluation; drug administration was initiated when P2 tumors reached 100–150 mm³ (2–4 weeks after P2 transplantation), with the timing adjusted by tumor biology, mouse strains, and transplantation procedures.

The tumor size was measured once or twice a week, and the tumor volumes were determined using the following equation: *V* (mm^3^) = (length × width²)/2. The mice were administered with trametinib (2 mg/kg, p.o., five times weekly), flavopiridol (5 mg/kg, i.p., five times weekly), or AZD8055 (20 mg/kg, p.o., five times weekly) as single agents or in combination (Tram/Flav, Tram/AZD, or Gem/PTX; gemcitabine 10 mg/kg, i.p., twice weekly; paclitaxel 20 mg/kg, i.p. once weekly). Vehicle-treated mice were used as controls for four consecutive weeks.

### Statistical analysis

All statistical calculations were performed using unpaired Student’s *t*-test or two-way ANOVA using GraphPad Prism version 9.0. The statistical details were indicated in the figures or associated legends.

## Results

### PDOs preserve the morphological characteristics and genetic alterations

**Figure 1:**
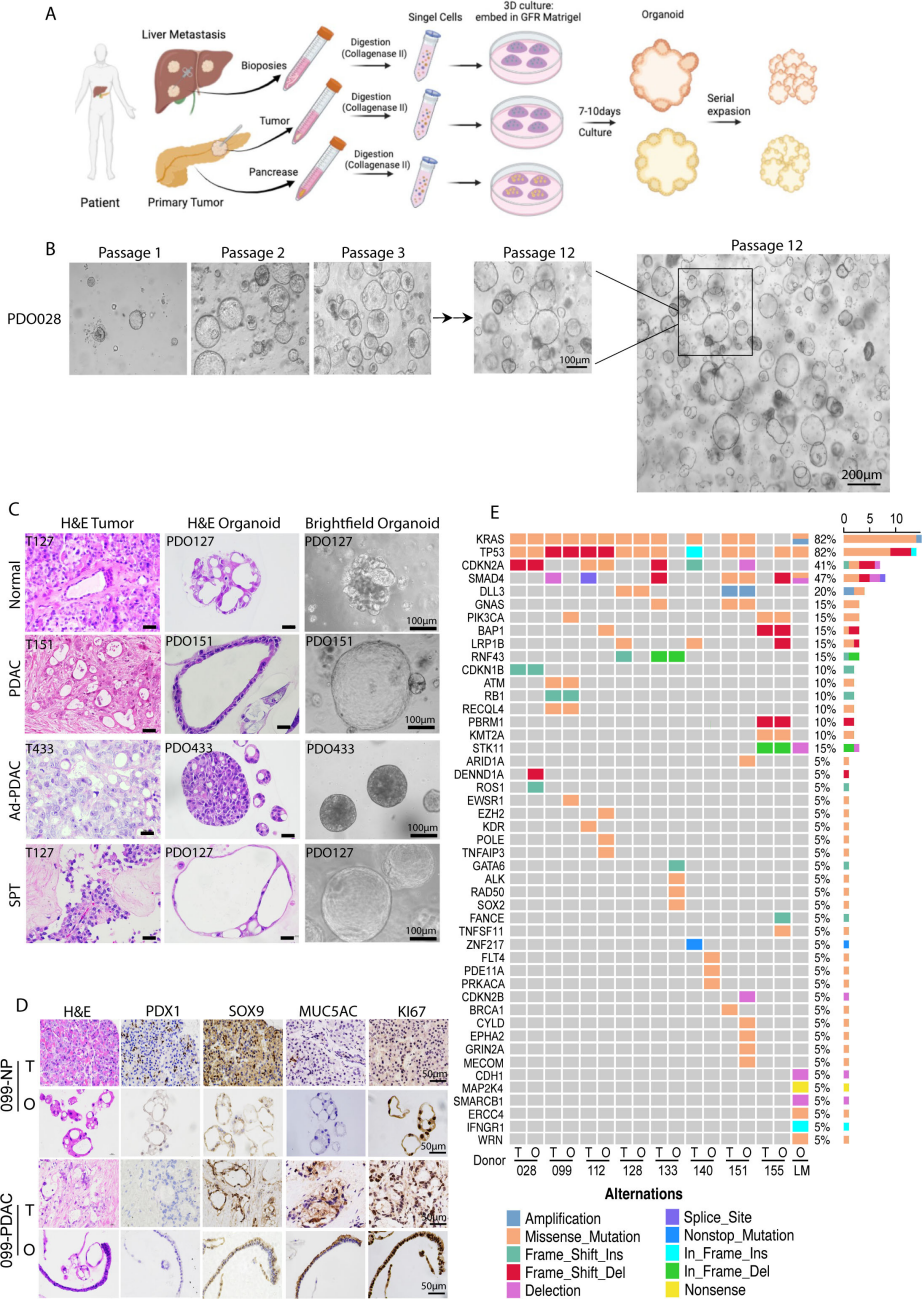
Establishment and long expansion of pancreatic adenocarcinoma organoids derived from human surgical tissues or biopsies. **(A)** Schematic depiction of PDO generation and expansion. **(B)** Representative images showing 028 tumor organoid expansion in 12 passages, maintaining stable lumen morphology. **(C)** Brightfield images of H&E staining of human tissues and corresponding organoids. **(D)** The expression of key genes involved in PDX1, SOX9, MUC5AC, and KI67 was detected by IHC. **(E)** A sequencing panel of 425 oncogenic genes identified somatic mutations in PDAC tissues and the corresponding organoids, with normal tissues and organoids as negative controls, respectively.

We collected 66 pancreatic tissue samples (39 tumors, 27 normal) from patients at the First Affiliated Hospital of Xi’an Jiaotong University. The tumor subtypes included pancreatic ductal adenocarcinoma (PDAC; 21/39), pancreatic ductal adenocarcinoma with liver metastasis (PDAC-LM; 1/39), solid pseudopapillary tumor of the pancreas (SPT; 4/39), pancreatic neuroendocrine tumor (PNET; 2/39), and others. The PDX models were established from 32 tumors, with a 31% success rate (10/32) (**Supplementary Tables 1**, **2**). PDOs were established from normal and tumor tissues collected during surgical resection or biopsy to create an organoid biobank. Fresh tissues were enzymatically digested into single-cell suspensions and embedded in growth-factor-reduced Matrigel. These were subsequently overlaid with HPO complete medium containing a cocktail of growth factors and small molecule inhibitors (as detailed in “Materials and methods”) (**Figure 1A**), achieving success rates of 69% (27/39) for tumors and 54% (14/27) for normal tissues. Among tumor PDOs, 21 were PDAC-derived, one was from PDAC-LM, and five were from non-PDAC subtypes (**Supplementary Figure 1A**; **Supplementary Table 1**). The PDO morphology varied from cystic to dense structures. PDAC-LM PDOs were dense (**Supplementary Figure 1B**). Organoids derived from tumor or adjacent normal tissue from the same PDAC patient revealed no morphological differences (**Supplementary Figure 1C**) and remained stable across passages (passages 1 to 12) (**Figure 1B**). In summary, our culture system facilitates the generation of organoids from tissues, allowing for long-term expansion.

H&E staining confirmed that PDOs recapitulated primary tumor histology (**Figure 1C**). To further characterize the cellular composition of the organoids, we assessed the expression of pancreatic lineage markers—PDX1, a marker for pancreatic progenitors and beta cells, and SOX9, a ductal marker. MUC5AC, which is uniquely expressed in pancreatic cancer, serves as a diagnostic marker (18). Ki67 has been extensively utilized as a proliferation marker for several decades, serving as an indicator of tumor proliferation rate. Immunostaining revealed the expression of pancreatic markers (PDX1 and SOX9) and tumor-specific markers (MUC5AC and Ki67) (**Figure 1D**; **Supplementary Figure 2A**). Epithelial markers (CK7, CK20, and CDX2) were also preserved (**Supplementary Figure 2B**).

The NGS of 425 oncogenes in eight PDAC PDO–tumor pairs and one LM-PDO showed KRAS mutations in 2/3 lines (028, 099, 112, 128, 151, 433-LM) and TP53 mutations in 5/8, with additional alterations in CDKN2A, SMAD4, and DLL3. PDOs retained most parental tumor mutations but acquired new variants during culture (**Figure 1E**).

Overall, PDOs faithfully mimic the pathological and genetic features of pancreatic tumors, enabling long-term expansion and study.

### PDOs exhibit comparable drug responses in both early and late passages alongside pharmacodynamic heterogeneity

**Figure 2:**
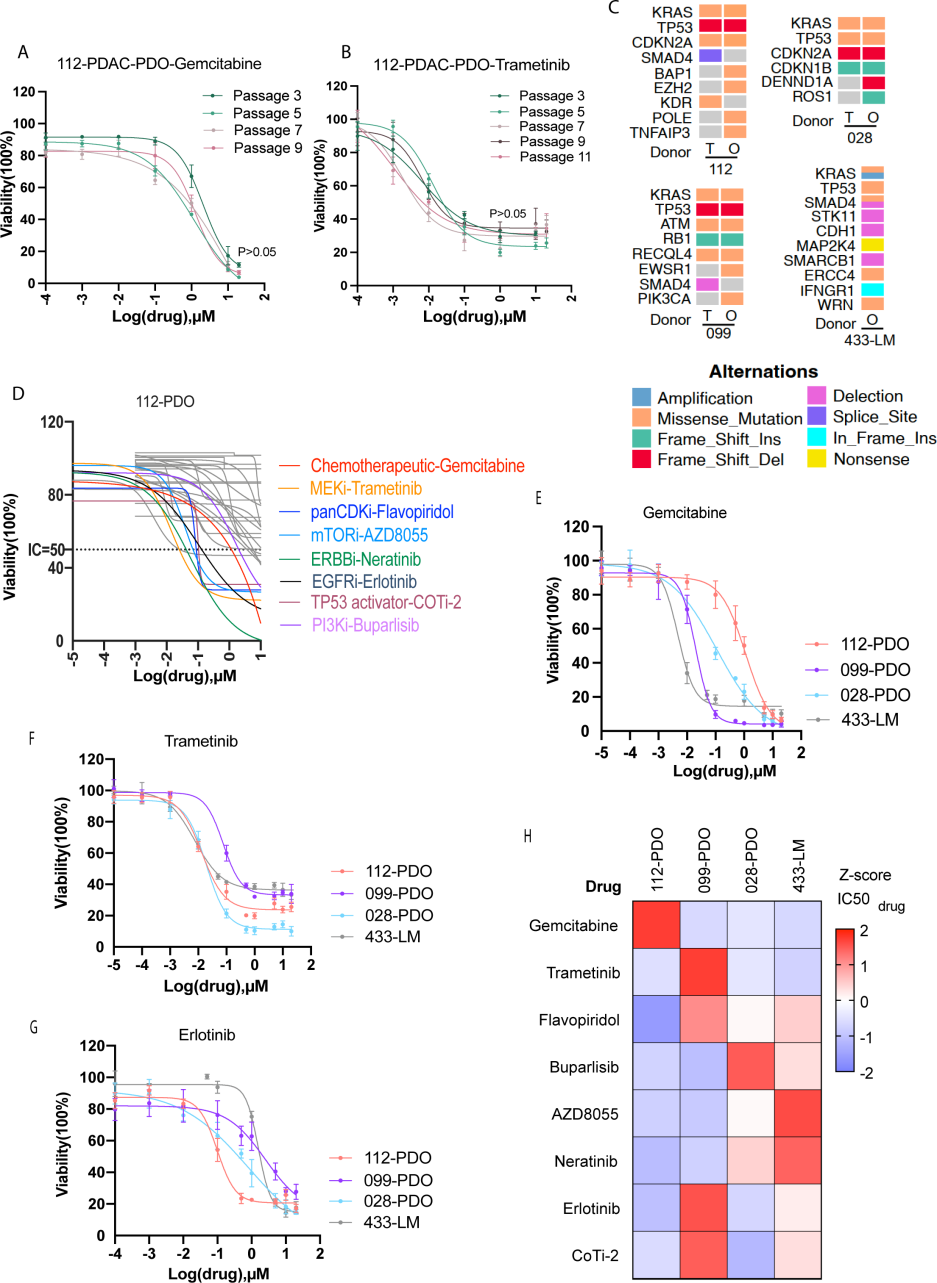
PDOs exhibit pharmacodynamic heterogeneity. **(A, B)** Dose–response curves of early and late PDAC organoids treated with gemcitabine **(A)** and trametinib **(B)**, indicating similar growth inhibition among different passages. Data are mean ± s.d., *n* = 4 per each group, Two-tailed paired *t*-test. **(C)** Genomic landscape of four PDO, including PDAC (028, 099, and 112) and PDAC-liver metastasis (433-LM). **(D)** Dose–response curves of PDO112 exposed to a total of 32 compounds, including stand-of-care chemotherapeutics and drugs targeting the pathway associated with mutant genes. The gray curves indicate resistance; the colorful curves indicate sensitivities. **(E)** For PDO112, 099, 028, and 433-LM, varied drug response rates to chemotherapeutics–gemcitabine were observed. **(F, G)** For PDO112, 099, 028, and 433-LM, varied drug response rates to trametinib–MEK inhibitor **(F)** and erlotinib–EGFR inhibitor **(G)** were observed. **(H)** Response of four PDOs to eight effective drugs targeting the biological process or pathway involved in tumor initiation and progression. The z-scores of the obtained IC50 values are depicted in the heatmap.

To explore the potential application of our PDO models in characterizing drug sensitivity signatures, we expanded four PDO lines that effectively retained the genetic mutations of their tumor origins for further investigation. Initially, we assessed the sensitivity of PDOs to gemcitabine (first-line chemotherapeutic agent) and trametinib (MEK inhibitor), Consistent responses to gemcitabine and trametinib were observed across passages (**Figures 2A, B**). Genomic analysis confirmed KRAS/TP53 mutations in all lines, with CDKN2A mutations in PDO112/028 (**Figure 2C**).

To elucidate the drug sensitivity and pharmacodynamic heterogeneity signatures of PDAC organoids, a 32-drug screen on PDO112 identified eight active agents (gemcitabine, trametinib, flavopiridol, AZD8055, neratinib, erlotinib, COTI-2, and buparlisib) with >50% inhibition (**Figure 2D**; **Supplementary Figure 3A**). Dose–response testing in three additional PDOs revealed variable sensitivity patterns (**Figures 2E–G**; **Supplementary Figure 3B**), visualized in an IC50 heatmap, with red indicating resistance (high values) and blue indicating sensitivity (low values) (**Figure 2H**). These results highlight significant inter-patient heterogeneity in drug responses.

### Identification of synergistic drug combinations that enhance the efficacy of MEK inhibitor therapy

While MEK inhibitors show limited efficacy against KRAS-driven PDAC due to pathway reactivation (10, 19–23), we identified synergistic combinations through secondary screening. Trametinib (MEKi) was paired with six targeted agents, with AZD8055 (mTORi) and flavopiridol (pan-CDKi) showing the most promise (Bliss analyses were conducted to determine whether the inhibitory activities of each combination were additive or synergistic, with Bliss scores greater than 1.0 indicating synergy).

**Figure 3:**
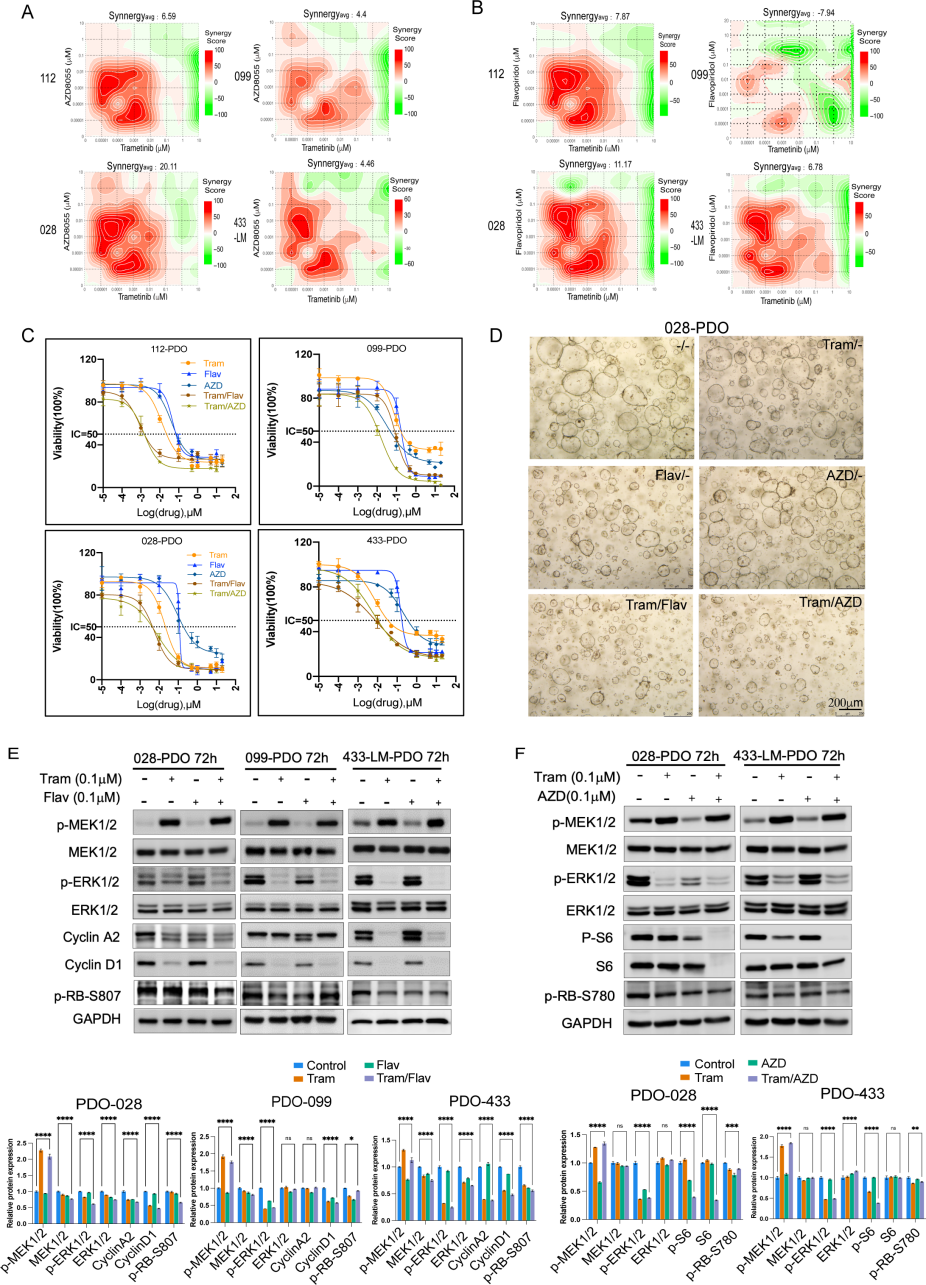
Discovery of synergistic drug combinations that enhance the MEKi therapy. **(A)** The representative bliss synergy score heatmap of MEKi combination with MTORi is shown. Red, synergy; green, antagonism; white, no effect. The averaged dose–response curves of three independent experiments (with three technical replicates) are shown. Synergy_avg_, average bliss synergy score. **(B)** The representative bliss synergy score heatmap of the MEKi combination with Pan-CDKi is shown. Red, synergy; green, antagonism; white, no effect. The averaged dose–response curves of three independent experiments (with three technical replicates) are shown. Synergy_avg_, average bliss synergy score. **(C)** Dose–response curves of four PDOs treated with MEKi alone or in combination with pan-CDKi or MTORi for 96 h. Cell viability was measured by using CellTiter-Glo^®^ 3D Cell Viability Assay. Data are mean ± s.d. **(D)** Representative images of PDAC organoid 028 treated with the vehicle, trametinib, flavopiridol, AZD8055 alone, or a dual combination for 72 h. **(E)** PDO028, PDO099, and PDO433-LM were treated with the indicated drugs for 72 h. Cell lysates were immunoblotted to determine the levels of phosphorylated and total MEK, ERK, cyclin A2, cyclin D1, phosphorylated RB, and the loading control GAPDH. **(F)** PDO028 and PDO433-LM were treated with the indicated drugs for 72 h. The cell lysates were immunoblotted to determine the levels of phosphorylated and total MEK, ERK, S6, phosphorylated RB, and the loading control GAPDH. Error bars indicate mean ± SEM; *P < 0.05, **P < 0.01, ***P < 0.001, ****P < 0.0001; two-way ANOVA test.

In this study, we concentrated on two MEK inhibitor (MEKi) combinations: one with AZD8055, an mTOR inhibitor (MTORi), a next-generation ATP-competitive pan-mTOR inhibitor, to replace the first-generation rapalogs used in previous studies that only partially inhibit mTORC1. AZD8055 can simultaneously target mTORC1 and mTORC2, with significantly improved target affinity and specificity, thereby strengthening the pathway inhibitory effect of the combination. Our findings indicate that the simultaneous inhibition of trametinib and AZD8055 significantly enhanced the inhibitory effect across four organoid lines. The Tra/AZD combination demonstrated strong synergy across all four PDO lines (Bliss scores: 6.59, 4.4, 20.11, and 4.46; **Figure 3A**). The other was with flavopiridol, a pan-CDK inhibitor (pan-CDKi). Tra/Flav showed synergy in three lines but failed in PDO099 (Bliss score: -7.94; **Figure 3B**). The dose–response curves confirmed enhanced growth inhibition with both combinations (**Figure 3C**), supported by organoid imaging (**Figure 3D**; **Supplementary Figure 4**). Western blot analysis demonstrated that treatment with Tra/Flav resulted in significantly decreased protein levels of pERK, cyclin D1, and pRB relative to the control group, whereas cyclin A2 expression in PDO099 showed no such reduction (**Figure 3E**). Tra/AZD significantly suppressed pERK, pS6, and pRB (**Figure 3F**).

### Validating synergistic combinatorial MEKi therapies *in vivo* using PDX models derived from the same patient

**Figure 4:**
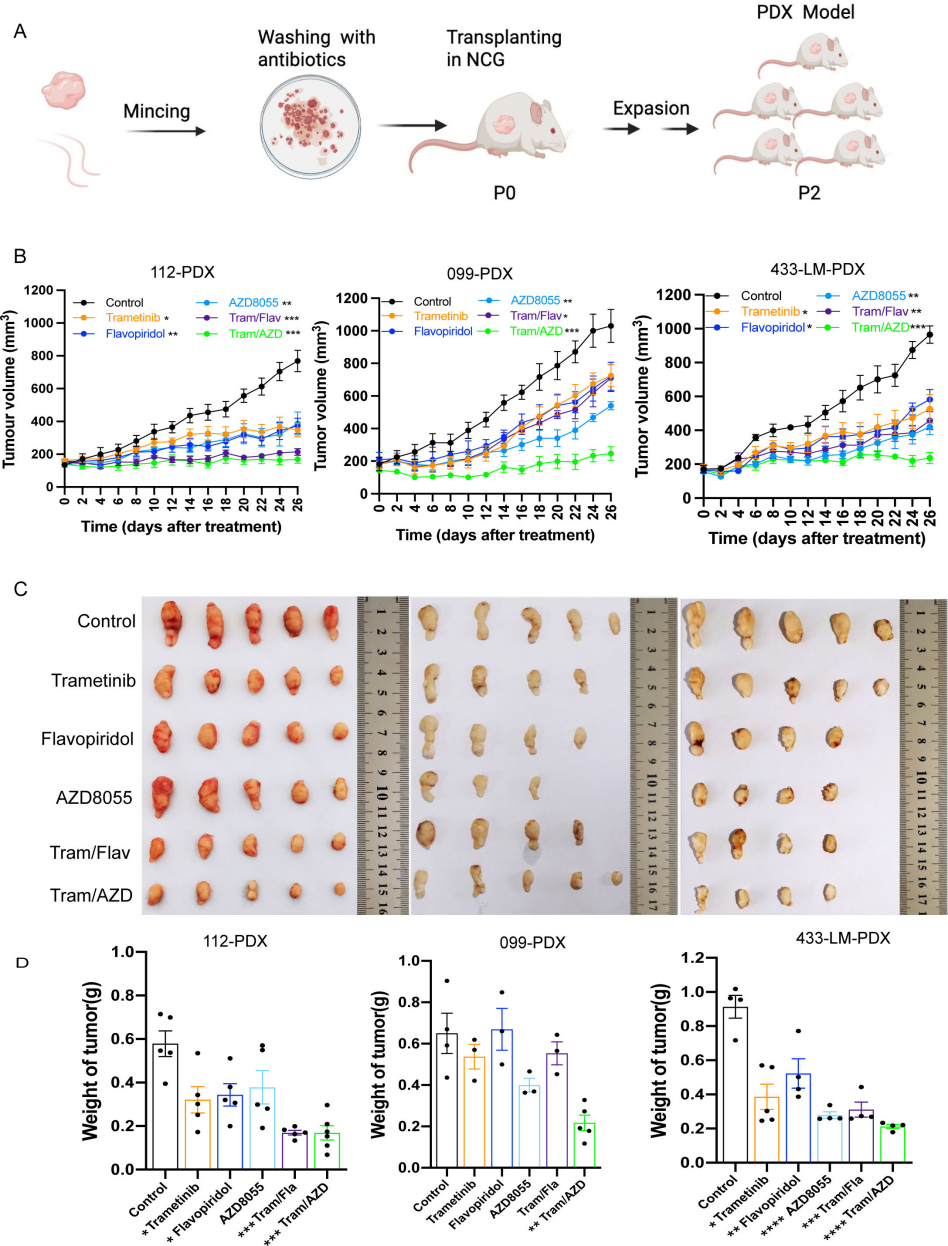
Validating synergistic combinatorial MEKi therapies *in vivo* using PDX models derived from the same patient. **(A)** Schematic illustration of the establishment of PDX. **(B)** Tumor volumes of mice bearing PDAC tumors (PDX112, PDX099, PDX433-LM) treated with vehicle, Tram, Flav, AZD alone, or a dual combination for the indicated times. **(C)** Tumor images of NCG mice bearing PDAC tumors following a 4-week treatment as indicated (*n* = 5 per group). **(D)** Tumor weight of mice bearing PDAC tumors (PDX112, PDX099, PDX433-LM) treated as indicated (error bars indicate mean ± SEM; *n* = 5 per group; *P < 0.05, **P < 0.01, ***P < 0.001, ****P < 0.0001; unpaired Student’s *t*-test).

We assessed the synergistic antitumor activity of trametinib-based combinations (Tram/Flav and Tram/AZD) in four PDX models (PDX-112, PDX-099, PDX-433LM, and PDX-028). These PDX models and their paired PDOs were derived simultaneously from the same patient-derived tumor specimens (**Figure 4A**). When the tumor volume of P2 reaches 100–150 mm^3^, the mice were randomized to seven treatment groups (*n* = 5/group): vehicle control, single agents (trametinib—2 mg/kg, flavopiridol—5 mg/kg, and AZD8055—20 mg/kg), and both combinations, administered 5×/week for 28 days. The tumor growth and body weight of the mice were monitored throughout the treatment period (**Figure 4B**; **Supplementary Figures 5A–C**). At the end of the study, the tumors were harvested and weighed (**Figures 4C, D**; **Supplementary Figures 5B, C**). Tram/AZD had significantly reduced tumor volume in all PDXs, while Tram/Flav showed efficacy in three PDXs but failed in RB-mutant PDX-099, mirroring the PDO results. There was no significant decrease in mouse body weight, and the overall tolerability was acceptable.

### Prediction of the therapeutic response to gemcitabine/paclitaxel in PDAC both *in vitro* and *in vivo*

To verify the predictive value of patient-derived organoid (PDO) and paired patient-derived xenograft (PDX) models for PDAC patients’ clinical therapeutic responses to drugs, we selected the gemcitabine/paclitaxel (Gem/PTX) combination chemotherapy regimen [a standard PDAC regimen (24)] for *in vitro* and *in vivo* efficacy validation. This regimen was determined based on the complete follow-up of the clinical treatment courses of the key enrolled patients (patient 099 and patient 112). Neither patient received any targeted drug therapy nor the fluorouracil + leucovorin + irinotecan + oxaliplatin (FOLFIRINOX) chemotherapy regimen during clinical diagnosis and treatment. Specifically, patient 099 was given Gem/PTX combination chemotherapy as the second-line treatment after disease progression following the first-line Gem/Tegafur therapy, while patient 112 received Gem/PTX as the core therapeutic regimen directly in clinical practice. The Lewis carbohydrate antigen 199 (CA19-9) is extensively utilized as a diagnostic and prognostic biomarker for pancreatic cancer (25). Serum CA19–9 levels are instrumental in evaluating the impact of neoadjuvant therapy in patients with pancreatic cancer (26).

**Figure 5:**
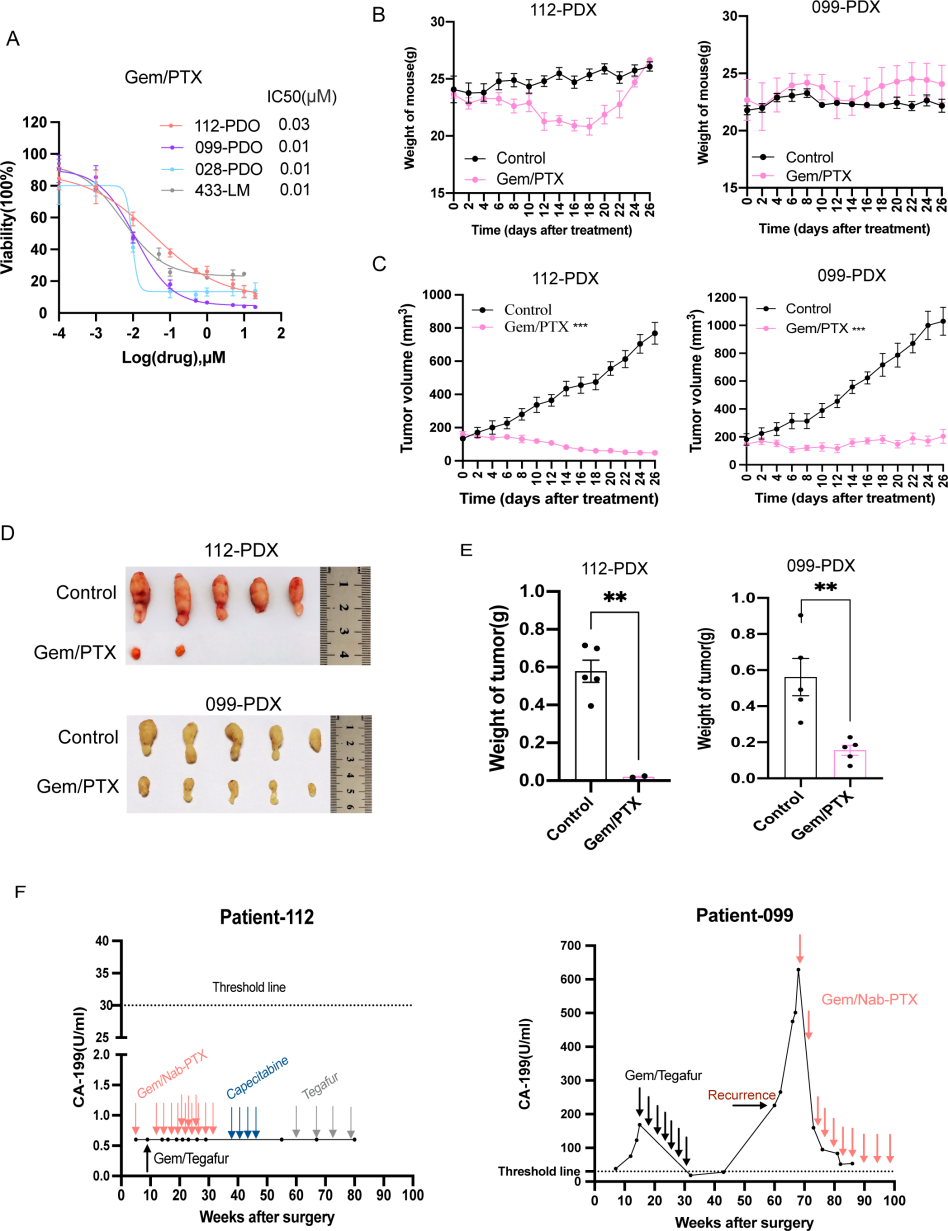
Prediction of the clinical therapy response of Gem/PTX in human PDAC models *in vitro* and *in vivo*. **(A)** *In vitro*, dose–response curves of four PDOs treated with Gem/PTX for 96 h. Cell viability was determined. Representative results from three independent experiments are shown (*n* = 4, mean ± s.d.). **(B–E)** *In vivo*, tumor growth inhibition was observed in PDX models (PDX-112, PDX-099) treated with vehicle (“control”), Gem/PTX, for 26 days. Weight curves of mice during experiments **(B)**, growth of tumor volume curves **(C)**, tumor images of mice at the end of the experiments **(D)**, and weight of tumor at the end of the experiments **(E)**. **(F)** CA199 levels of the indicated patients after accepting the Gem/Nab-PTX therapy. In this part, the control group tumors were the same as those shown in **Figure 4** because these animals experiments were conducted during the same period. Error bars indicate mean ± SEM; *n* = 5 per group; **P < 0.01, ***P < 0.001; unpaired Student’s *t*-test.

In this study, four PDO lines (112, 028, 099, and 433) showed 75%–95% growth inhibition after 96 h of treatment (**Figure 5A**). The corresponding PDX models (PDX-112 and PDX-099) confirmed these results *in vivo*, with Gem/PTX (50 and 20 mg/kg, respectively) significantly reducing the tumor growth over 28 days. Mice weight and tumor size were measured bi-daily (**Figures 5B, C**). Tumor images and weights were collected at the end of treatment (**Figures 5D, E**). CA19–9 monitoring (cutoff: 30 U/mL) revealed favorable responses in patient 099 and patient 112 (**Figure 5F**), mirroring clinical outcomes. Gemcitabine and paclitaxel (Gem/PTX) significantly inhibited tumor growth both *in vivo* and *in vitro*, aligning with clinical responses observed in patients. This PDO-to-PDX concordance demonstrates the PDO models’ reliability in predicting drug responses.

The results of this study showed that the drug sensitivity test results of the PDO model for Gem/PTX were effectively verified in the paired PDX models and were highly consistent with the clinical therapeutic responses of patient 099 and patient 112, which suggests that the PDO/PDX models have the potential to predict PDAC patients’ therapeutic responses to chemotherapy regimens practically applied in clinical settings.

## Discussion

Pancreatic ductal adenocarcinoma (PDAC) remains a highly lethal malignancy with profound intratumoral and interpatient heterogeneity, which severely impedes the development of effective targeted and chemotherapeutic strategies. Patient-derived organoids (PDOs) and patient-derived xenografts (PDXs) have emerged as transformative preclinical models that recapitulate the genomic, morphological, and functional features of primary tumors, bridging the gap between *in vitro* cell line studies and clinical patient outcomes. In this study, we established a well-characterized PDAC biobank comprising 27 tumor PDOs (69% success rate) and 10 matched PDXs (31% success rate) from surgical and fine-needle biopsy specimens, alongside 14 normal pancreatic PDOs. Our findings validate the robust fidelity of these models in preserving the pathological, genetic, and pharmacodynamic characteristics of parental tumors and further demonstrate their utility in identifying synergistic targeted combinations and predicting clinical chemotherapy responses for KRAS-mutant PDAC—the most prevalent and clinically challenging subtype of pancreatic cancer.

KRAS mutation is the core oncogenic driver of over 90% of PDAC cases, and the intractability of directly targeting mutant KRAS has led research efforts to focus on inhibiting its downstream effector pathways, among which the MAPK pathway is one of the most well-characterized and clinically explored. As a key regulatory node in the MAPK pathway, MEK has become an important targeted therapeutic candidate for KRAS-mutant PDAC, yet single-agent MEK inhibitors have been found to yield limited clinical efficacy in this malignancy, a shortcoming largely attributed to adaptive pathway reactivation and the extreme molecular and phenotypic heterogeneity of PDAC. To address this, we performed a 32-drug screen on PDAC PDOs and identified eight agents with significant growth-inhibitory activity, subsequently conducting rational combination screening centered on the MEK inhibitor trametinib. Our results revealed that the combination of trametinib with the next-generation pan-mTOR inhibitor AZD8055 exerted robust synergistic antitumor effects across all four tested PDO lines (Bliss synergy scores 4.4–20.11) and their matched PDX models. This synergy is not a novel pathway-level discovery but rather a clinically relevant validation of the MEK/mTOR combination in patient-derived models—an improvement over previous studies that relied on immortalized cell lines or genetically engineered mouse models. Critical to this improvement is our selection of AZD8055, which simultaneously targets mTORC1 and mTORC2 with high specificity, replacing first-generation rapalogs that only partially inhibit mTORC1 and exhibit limited efficacy. Additionally, unlike the non-selective pan-pathway combination strategies of prior research, our study employed a sequencing-guided personalized workflow—integrating 425-gene-panel genomic profiling, single-agent drug screening, and rational combination testing—greatly enhancing the clinical pertinence of our findings. Mechanistically, the MEK/mTOR combination directly targets the core downstream effectors of the KRAS signaling network, avoiding the pathway compensation that limits the efficacy of MEK/PI3K combinations by circumventing the PI3K-inhibition-induced relief of mTORC1 negative feedback, thus achieving more complete blockade of oncogenic signaling in KRAS-mutant PDAC.

In contrast to the universal synergy of the trametinib/AZD8055 combination, the trametinib/flavopiridol (pan-CDK inhibitor) combination failed to elicit a synergistic effect in PDO-099 and its matched PDX-099, a finding that highlights the molecular heterogeneity underlying variable drug responses in PDAC. While initial correlative data suggested a potential association between RB1 status and this resistance, functional validation of this link was not performed in the current study; thus, we refrain from drawing definitive conclusions regarding RB1 mutation-mediated resistance to the MEK/CDK inhibitor combination. This observation, however, underscores the importance of functional drug testing in patient-derived models to complement genomic profiling, as genetic alterations alone may not fully predict therapeutic responses.

Beyond targeted therapy, we validated the predictive value of PDO models for clinical chemotherapy responses using the gemcitabine/paclitaxel (Gem/PTX) combination—selected based on the actual clinical treatment regimens of our enrolled patients. Four PDAC PDO lines exhibited 75%–95% growth inhibition in response to Gem/PTX, and this efficacy was recapitulated in matched PDX models derived from the same patients. Most notably, the *in vitro* drug sensitivity of PDO-099 and PDO-112 was highly consistent with the clinical responses of the corresponding patients, as evidenced by the reduced serum CA19–9 levels and clinical remission following Gem/PTX treatment. For patient 099, who progressed on first-line gemcitabine/tegafur therapy but responded to second-line Gem/PTX, the PDO model preserved the intrinsic clonal heterogeneity of the primary tumor, including pre-existing gemcitabine/tegafur-resistant clones with no cross-resistance to Gem/PTX. This finding not only confirms the ability of PDOs to capture regimen-specific drug resistance in PDAC but also demonstrates their potential to guide chemotherapy optimization for individual patients—an especially valuable feature given the lack of reliable predictive biomarkers for PDAC chemotherapy.

The high concordance in drug response between PDOs and their matched PDXs further bolsters the reliability of PDO-derived drug efficacy data, as we conducted a comprehensive preclinical drug evaluation using both models sourced from the same patient, thereby minimizing variability in drug efficacy attributable to individual patient differences. PDOs themselves maintain stable drug sensitivity profiles across early and late passages and retain the majority of parental tumor genetic alterations, making them a reliable and sustainable platform for long-term pharmacodynamic studies. What is more, the *in vivo* verification from PDXs lends robust preclinical credence to the PDO-based drug sensitivity and synergy findings, which, in turn, strongly supports the great clinical translational potential of PDO models. While PDX models serve as a valuable auxiliary tool for *in vivo* drug efficacy verification, they suffer from inherent drawbacks that limit their practical application in clinical translation—including a markedly lower modeling success rate as well as the time-consuming and high-cost nature of their establishment and maintenance, in sharp contrast to the efficient, low-cost, and high-success-rate characteristics of PDOs that make them well suited for clinical translational research and personalized therapy application.

Collectively, our study establishes a clinically relevant PDO biobank for KRAS-mutant PDAC and identifies the trametinib/AZD8055 combination as a promising targeted therapy strategy with robust synergistic activity in PDO models. More importantly, we demonstrate that PDO models can accurately predict clinical chemotherapy responses, providing a practical and functional tool for personalized therapy in PDAC—a disease where genomic profiling alone is often insufficient to guide treatment decisions. These findings lay a foundation for the further development of personalized targeted and chemotherapeutic strategies for KRAS-mutant PDAC and highlight the prominent value of PDO models in early-phase drug development and clinical treatment decision-making, underscoring their great potential for clinical translation in the management of PDAC.

### Limitations

This study provides valuable preclinical evidence for the clinical translational potential of patient-derived organoid (PDO) models in the personalized therapy of KRAS-mutant pancreatic ductal adenocarcinoma (PDAC), yet several inherent limitations persist. These research shortcomings also point to the core directions for our subsequent in-depth investigations into PDO-based PDAC therapeutic strategies.

Synergistic drug combination screening was only conducted on four PDAC PDO lines, and the small cohort size severely restricts the generalizability of our findings and the establishment of robust drug response predictive signatures, with the resulting data serving merely as a preliminary exploratory analysis of PDO-based drug sensitivity prediction that cannot be directly extrapolated to guide clinical treatment decisions. Our established PDO models consist solely of epithelial cells and lack the desmoplastic stromal components that are a pathological hallmark of PDAC; this intrinsic defect not only risks introducing bias into *in vitro* drug efficacy assessments but also prevents the reflection of stromal regulation of drug efficacy and toxicity within the *in vivo* tumor microenvironment. In terms of drug toxicity evaluation, observations in PDX models were limited solely to mouse body weight monitoring, with no systematic organ-specific toxicity assessments—such as serum biochemical tests and histopathological analyses of key organs—performed. This incomplete evaluation approach means that the actual clinical adverse effect profiles of the tested drug combinations cannot be fully and authentically reflected. Genomic profiling of tumors and PDOs relied on a 425-cancer-related-gene panel, which effectively covers common oncogenic driver mutations but fails to capture rare driver mutations and structural variants associated with drug resistance; additionally, we did not conduct PDO-based functional drug screening for patients with no detectable actionable mutations, leaving a gap in the application of PDO models for this clinically refractory patient subgroup. We also lack longitudinal tumor sampling from patients during disease progression and the construction of serial PDO models from post-progression tumor tissues, which hinders the direct verification of clonal evolution in drug-resistant populations and the in-depth exploration of the molecular mechanisms underlying regimen-specific drug resistance in PDAC. Furthermore, the PDX models used for *in vivo* validation were established in immunodeficient NCG mice, precluding the evaluation of the immunomodulatory effects of the tested drug combinations and the investigation of interactions between targeted therapy and the host anti-tumor immune response—a key research direction for the development of combined anti-tumor strategies in the future.

We seek to address these limitations in a targeted manner in our follow-up research, where we plan to adopt a series of measures including optimizing the study design and refining the model system. These efforts aim to gradually enhance the scientific rigor and clinical relevance of our research findings in an effort to lay a more solid research foundation and accumulate valuable empirical evidence for the ongoing exploration of PDO models’ clinical translation in the personalized therapy of KRAS-mutant PDAC.

## Data Availability

All sequencing data and supporting materials generated and analyzed in the present study are available upon request to interested researchers. To access these materials, researchers are kindly requested to submit a reasonable application to the corresponding author, who will provide detailed access guidelines to ensure the reproducibility and transparency of the research outcomes. If readers are interested in the sequencing data, they may send an email to indicate their intention, and we will provide the detailed data accordingly.
