# Supplementary material for: Patient-derived organoids guide personalized therapy for KRAS-mutant pancreatic cancer: synergistic MEK/mTOR inhibition and predictive chemotherapy responses
Source: Front Immunol. 2026 May 15;17:1760379. doi: 10.3389/fimmu.2026.1760379 (PMC13218876; doi:10.3389/fimmu.2026.1760379)
Supplement: Supplementary file 1 [file Table1.docx]

Supplementary Tables

Supplementary Figure and Legend

Supplement figure 1

(A) Pie chart depicting the characteristics of PDO which established successfully in this work.

(B)Brightfield images of 4 PDO derived from normal pancreas donor (PDO128), pancreatic ductal adenocarcinoma donor (PDO028), solid pseudopapillary tumor of pancreas donor (PDO189) and pancreatic ductal adenocarcinoma liver metastasis donor (PDO433). Representative images of each PDO forming.

(C) Brightfield images of 2 PDO from the same patient’s normal pancreas donor and PDAC donor tissues indicating the grown of organoids.

Supplement figure2

(A) Expression of key genes involved in pancreatic fate (PDX1), ductal fate (SOX9), cancer marker (MUC5AC) and proliferation marker (KI67) were detected by immunohistochemistry on organoids and original tissues.

(B) Expression of key genes involved in epithelial cells

Supplement figure3

(A) Chemotherapy drugs in NCCN and targeted drugs associated mutated genes used in this work.

(B) Dose-response curves of 4 indicated PDOs treated with drugs screening out in(F). Data are mean ± s.d., n=4 per each group.

Supplement figure 4

Representative images of PDAC organoid 099 treated with the vehicle control DMSO(-) or MEKi(Tra/-), Pan-CDKi(Fla/-), MTORi(AZD/-), MEKi/Pan-CDKi(Tra/Fla), MEKi/MTORi (Tra/AZD).

Supplement figure 5

(A)The weight of mice bearing PDAC tumors treated with vehicle (“Control”), trametinib(“Tram”), flavopiridol(Flav), AZD8055(AZD), or Tram/Flav,Tram/AZD for indicated times (mean ± SEM; n =5per group; *P < 0.05, **P < 0.01, ***P < 0.001, ****P < 0.0001; unpaired Student’s t test).

(B)In vivo PDX028 models validated the synergistic combinations of MEKi and Pan-CDKi to suppress the tumor growth(mean ± SEM; n =5per group; *P < 0.05, **P < 0.01, ***P < 0.001, ****P < 0.0001; unpaired Student’s t test).

(C) In vivo PDX028 models validated the synergistic combinations of MEKi and MTORi to suppress the tumor growth(mean ± SEM; n =5per group; *P < 0.05, **P < 0.01, ***P < 0.001, ****P < 0.0001; unpaired Student’s t test).
