# Supplementary material for: Patient-derived organoids guide personalized therapy for KRAS-mutant pancreatic cancer: synergistic MEK/mTOR inhibition and predictive chemotherapy responses
Source: Front Immunol. 2026 May 15;17:1760379. doi: 10.3389/fimmu.2026.1760379 (PMC13218876; doi:10.3389/fimmu.2026.1760379)
Supplement: Supplementary file 2 [file Table2.docx]

Figure 3E

Figure 3F
